# EvSec22, a SNARE Protein, Regulates Hyphal Growth, Stress Tolerance, and Nematicidal Pathogenicity in *Esteya vermicola*

**DOI:** 10.3390/jof11040295

**Published:** 2025-04-09

**Authors:** Jingjie Yuan, Run Zou, Xuan Peng, Yilan Wang, Zhongwu Cheng, Tengqing Ye, Lihui Han, Chengjian Xie

**Affiliations:** The College of Life Science, Chongqing Normal University, Chongqing 401331, China; y18225267527@163.com (J.Y.); m13658474511@163.com (R.Z.); 13278697122@163.com (Z.C.); hanlihui999@163.com (L.H.)

**Keywords:** *Esteya vermicola*, biocontrol fungus, pathogenicity, *Bursaphelenchus xylophilus*, SNARE proteins

## Abstract

*Bursaphelenchus xylophilus*, the causative agent of pine wilt disease (PWD), poses a severe global threat to coniferous forests. *Esteya vermicola*, an endoparasitic nematophagous fungus, exhibits promising biocontrol potential against this pinewood nematode. The vesicular transport system, evolutionarily conserved in eukaryotes, is essential for fungal pathogenicity. Based on our genome sequence of *E. vermicola* CBS115803, we identified *EvSec22*, a gene encoding a SNARE protein implicated in vesicular transport process. This study investigates the role of EvSec22 in *E. vermicola* during nematode infection, utilizing our optimized gene knockout methodology. Infection assays revealed that *EvSec22* deletion significantly impaired the pathogenicity of *E. vermicola* against *B. xylophilus*. Phenotypic analyses revealed that the *ΔEvSec22* mutant exhibited suppressed hyphal growth, reduced conidiation, and abnormal septal spacing. Furthermore, the mutant showed significantly diminished tolerance to osmotic stress (sorbitol) and oxidative stress (hydrogen peroxide). Overall, the *EvSec22* gene is associated with the virulence of *E. vermicola* CBS115803 against *B. xylophilus*, and its deletion also affects the normal growth of *E. vermicola* and its tolerance to abiotic stress. This study providing new insights into SNARE protein functions in fungal biocontrol agents.

## 1. Introduction

The pine wood nematode (*Bursaphelenchus xylophilus*), originally identified in 1929 from declining *Pinus palustris* stands in Texas, USA [1], was subsequently established in 1972 as the causative agent of pine wilt disease (PWD), a coniferous tree epidemic commonly termed “pine tree cancer” due to its rapid lethality [2,3]. Globally, PWD is one of the most severe forest diseases, causing devastating damage to pine forest resources and ecosystems, particularly in East Asia, leading to significant economic losses [4,5,6]. Chemical nematicides are widely employed to manage *B. xylophilus*; however, their prolonged use frequently leads to diminished field efficacy, the development of nematode resistance, and significant environmental risks [7,8].

The use of fungi, bacteria, and actinomycetes as biological nematicides has gained increasing attention, particularly the development of nematophagous fungi for biocontrol applications [9,10]. *Esteya vermicola* is an endoparasitic fungus of *B. xylophilus* that can survive in pine trees and their resin secretions. This fungus attracts nematodes by releasing volatile compounds that mimic pine-derived chemicals. The lunate-shaped conidia of *E. vermicola* adhere to and subsequently infect *B. xylophilus*, forming penetration pegs that breach the nematode cuticle. Once inside, the fungus utilizes the nematode’s organic components for growth, eventually emerging as hyphae from the nematode carcass and producing new lunate-shaped conidia to continue the infection cycle [11]. Studies have shown that within 24 h, 90% of nematodes are infected by *E. vermicola* conidia, and complete nematode mortality occurs within 8–10 days, initiating a new infection cycle [11,12]. Due to its strong infection capability and endoparasitic nature, *E. vermicola* holds promise as a biocontrol agent against *B. xylophilus* [13,14]. Additionally, research indicates that culture media influence the production and infectivity of *E. vermicola* conidia. Since only lunate-shaped adhesive conidia possess infection capability, nutrient-rich potato dextrose agar (PDA) medium is suitable for large-scale fungal propagation, whereas nutrient-poor water agar (WA) medium is preferable for maximizing lunate-shaped conidia production [15].

In eukaryotic cells, most secretory proteins enter the endoplasmic reticulum (ER) lumen and are transported to the plasma membrane via vesicles or specialized carriers, progressing from the ER to the Golgi apparatus before reaching their target membranes [16]. SNAREs (soluble *N*-ethylmaleimide-sensitive factor attachment protein receptors) are a highly conserved class of membrane-associated proteins that play a vital role in exocytosis by facilitating vesicle–membrane fusion, a process crucial for maintaining cellular function and homeostasis [17]. Based on conserved amino acid residues within the SNARE motif, SNARE proteins are classified into v-SNAREs and t-SNAREs. v-SNAREs are typically embedded in transport vesicles, while t-SNAREs localize to target membranes [18]. In other types of cells or organisms, homologs of synaptic SNARE are involved in cytokinesis [19]. VAMP (also known as synaptobrevin) is a v-SNARE protein on the vesicle membrane, and in yeast, Snc1 and Snc2 are homologous proteins of VAMP, which are involved in the cytosolization of yeast secretory vesicles [20]. Syntaxin is a t-SNARE protein on the cytoplasmic membrane, and in yeast, Sso1p and Sso2p are homologous proteins of Syntaxin, which play a key role in the secretion process in yeast [21].

v-SNARE Snc1 is responsible for both paracrine and retrograde vesicular transport between the plasma membrane (PM) and the Golgi apparatus, and it interacts with the cytosolic SNARE (Sso 1/2, Sec9) and the cytosolic complex to complete the cytosolic process [22]. It has been demonstrated that the ER-Golgi transport cycle operates in a COPI (Coat Protein I)-dependent manner, requiring SNARE proteins to bind membranes via their transmembrane domains (TMDs) [23]. The conservation of the TMD in SNARE proteins highlights the significance of retrograde transport pathways in cellular function [24]. Studies have shown that the deletion of SNARE protein Sec22 impairs the extracellular secretion of virulence-associated proteins required for host infection. *VdSec22* in *Verticillium dahliae* regulates the secretion of various enzymes, such as cellulases, pectinases, and xylanases, which are involved in carbohydrate metabolism and host cell wall degradation. Loss of *VdSec22* reduces *V. dahliae* virulence in cotton [25]. Additionally, Sec22 contributes to effector secretion via exocytosis in *Colletotrichum orbiculare*, facilitating primary hyphal development [26]. *MoSec22* is also involved in *Magnaporthe oryzae* infection of rice. Deletion of *MoSec22* inhibits conidiation, disrupts cell wall integrity, and impairs reactive oxygen species (ROS) production [27]. Both of which are critical for hyphal germination, appressorium formation, hyphal tip growth, and pathogenicity [28,29].

Despite its established roles in various pathogenic fungi, the function of Sec22 in *E. vermicola* development and infection of *B. xylophilus* remains unexplored. This study investigates the role of EvSec22 in *E. vermicola* CBS115803 through gene knockout analysis.

## 2. Materials and Methods

### 2.1. Vector Construction and Fungal Transformation

The knockout vector was constructed following our previously established gene knockout protocol [30]. Upstream and downstream DNA fragments (1.5 kb each) of the *EvSec22* gene were amplified from the genome of *E. vermicola* CBS115803. A 2.0 kb hygromycin resistance gene cassette was amplified from the pSilent1 vector. Homologous recombination fragments were obtained via fusion PCR and then ligated into a linearized (*BamH* I) green fluorescent protein (GFP) expression plasmid (pKOXN) using a 2× Seamless Cloning Kit (D7010S, Beyotime Biotech Inc., Shanghai, China). The gene knockout plasmid (*pKOXN-EvSec22*) was introduced into *Agrobacterium tumefaciens* AGL1 via the freeze-thaw method. *E. vermicola* CBS115803 was transformed using *Agrobacterium*-mediated transformation, and knockout transformants were selected on a medium supplemented with 500 μg/mL cefotaxime and 200 μg/mL hygromycin B. The gene’s deleted strains were screened according to our published protocol. Detailed methods are provided in Supplementary Methods S1.

To construct the complementation vector, the full-length *EvSec22* gene, including its 1500 bp promoter and 1500 bp terminator sequences, was amplified using gene-com F/R primers and cloned into the complementation plasmid pMD-3. The resulting construct was introduced into the corresponding gene deletion strain (*ΔEvSec22*). Transformants were selected using cefotaxime at a concentration of 500 μg/mL, hygromycin B at 150 μg/mL, and Basta at 50 μg/mL. This study was further expanded to examine the expression levels of *EvSec22* gene using semi-quantitative RT-PCR. Total RNA was individually extracted using the Steady Pure Universal RNA Extraction Kit II (AG, Changsha, China), following the manufacturer’s instructions. Reverse transcription of the isolated RNA was then performed with All-In-One 5X RT MasterMix with gDNA Removal (Applied Biological Materials Inc., Richmond, BC, Canada). Expression of *EvSec22* was determined by RT-PCR. PCR cycling consisted of an initial step of denaturation at 94 °C for 2 min, followed by 25 cycles of 94 °C for 30 s, 57 °C for 30 s, and 72 °C for 30 s. The *E. vermicola β-Tubulin* gene served as the reference control in this analysis. The complementation strain was named *ΔEvSec22comp*. Primer sequences are listed in Table S1.

### 2.2. Infectivity Assay of B. xylophilus

To propagate the pine wood nematode, the *Botrytis cinerea* strain stored in the laboratory was inoculated onto PDA plates (Hope Bio-Technology, Qingdao, China) and incubated at 26 °C in a fungal incubator (MJX-250B, Changge Mingtu, Chengdu, China) until the fungal mycelium fully covered the entire 90 mm diameter plates (BKMAMLAB, Changsha, China). Subsequently, *B. xylophilus* nematodes were surface-sterilized by immersion in a 3% hydrogen peroxide solution and then inoculated onto *B. cinerea*. This process aimed to eliminate surface bacteria on the nematodes before infection experiments. After incubation at 26 °C for five days, nematodes were isolated for the subsequent *E. vermicola* infection assay.

Simultaneously, 50 μL of conidial suspension (5 × 10^6^ conidia) from wild-type *E. vermicola* CBS115803, mutant strains, and complemented strains were evenly spread onto WA plates. These 60 mm diameter plates were incubated in the dark at 26 °C for five days. A 20 μL suspension of *B. xylophilus* nematodes (~400 individuals) was then inoculated onto *E. vermicola* WA plates. Nematodes were observed every two days for up to 11 days using an inverted Motic AE31E optical microscope. Mortality rates were determined by counting the percentage of dead nematodes in the first 100 encountered [31]. Inoculation experiments of different *E. vermicola* strains on pine wood nematodes at each time point were repeated six times.

### 2.3. Conidia Count

A 5 μL suspension containing 1 × 10^7^ conidia from wild-type *E. vermicola* CBS115803, mutant strains, and complemented strains was spotted onto pre-prepared PDA plates and incubated at 26 °C for 14 days. Then, 2 mL of sterile water was used to wash the colonies. A sterile inoculation spatula was used to assist the elution process, and the conidial suspension was obtained by filtration through three layers of lens paper. The total number of lunate and rod-shaped conidia was counted using a hemocytometer (Qiujing, Shanghai, China) under a microscope (DM3000, Leica, Wetzlar, Germany), and the proportion of lunate conidia was calculated [32]. Each experiment was repeated three times.

### 2.4. Growth Assay on Solid Medium

A 5 μL suspension containing 1 × 10^7^ conidia from wild-type *E. vermicola* CBS115803, mutant strains, and complemented strains was spotted onto prepared 90 mm diameter PDA plates. After incubation at 26 °C for eight days, colony sizes were photographed and measured. Each experiment was repeated three times.

### 2.5. Hyphal Septal Distance Measurement

Wild-type *E. vermicola* CBS115803, mutant strains, and complemented strains were cultured on PDA plates at 26 °C for eight days. A 1:1 mixture of sterile water and Calcofluor White fluorescent dye was prepared, and hyphae were stained in this solution for 0.5 h. The hyphal septal distance was observed under a fluorescence microscope (DM3000, Leica, Wetzlar, Germany) [33]. Each experiment was repeated 25 times.

### 2.6. Abiotic Stress Assay

PDA solid media containing 0.7 M KCl, 1 mM H_2_O_2_, or 1 M sorbitol were prepared, with PDA serving as the control. A 3 μL conidial suspension was spotted onto the center of each medium [34]. The plates were then incubated upside down at 26 °C for 10 days. Photographs were taken of the colony, and the colony diameters were measured using ImageJ2 software (LOCI, University of Wisconsin, Madison, WI, USA). The measured colony diameters were analyzed, and relative growth inhibition (RGI) was used to assess stress tolerance; RGI = (DC − DT)/DC × 100%, where DC and DT denote the diameters of the colonies on control and stress plates, respectively [35]. Each experiment was repeated three times. The following is the recipe for PDA medium: 2 g of dextrose (for fungi, 2 g of sucrose for spore counting), 1.5 g of agar powder, and 20 g of potato boiled to softness; the potato filtrate was fixed to 100 mL and sterilized at 121 °C for 20 min. All disposable petri dishes were purchased from Life Science Research Products (Haimen, China). The fungus incubator model MJX-250B was used.

### 2.7. Data Analysis

The data were expressed as mean ± standard deviation (SD). Statistical analysis was performed using one-way analysis of variance (ANOVA) followed by Dunnett Test, utilizing GraphPad Prism 8.0 software (GraphPad, San Diego, CA, USA). The phylogenetic tree was constructed with the maximum likelihood method with MEGA11.0 software. The protein evolutionary model was analyzed using the “find best protein model”, resulting in LG + G, and bootstrap values were based on 1000 replicates. The colony diameter was measured using ImageJ2 (https://imagej.net/software/imagej2/ (accessed on 2 April 2025)) by first calibrating the image scale to ensure accurate size units then applying thresholding to isolate the colony from the background and finally using the measurement tools to extract the colony diameter data. All data processing steps ensured the accuracy and consistency of the measurements, providing reliable colony size information.

## 3. Result

### 3.1. Sequence Analysis of EvSec22 in E. vermicola CBS115803

**Figure 1:**
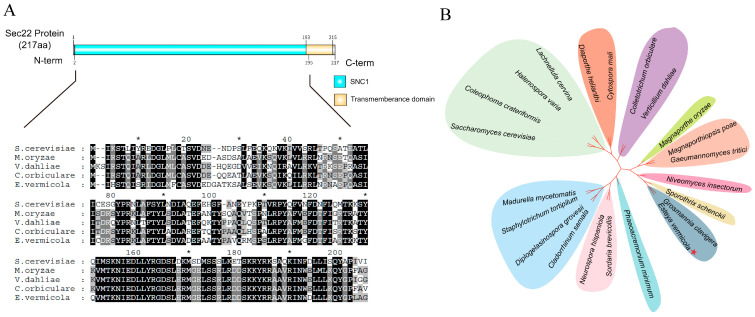
Homology comparison and phylogenetic analysis of EvSec22 amino acid sequences. (**A**) Multiple sequence alignment of EvSec22 with homologous amino acid sequences. For every 10 sites in the comparison results, add a black star. The intensity of the black shading reflects the level of amino acid similarity at each position, including sequences from *Saccharomyces cerevisiae* (DAA09582), *Magnaporthe oryzae* (KLU89933), *Verticillium dahliae* (XP_009658406), and *Colletotrichum orbiculare* (BAO27797). (**B**) Phylogenetic tree of Sec2*2* in *E. vermicola* and their homologs from the annotated NCBI protein database in other fungi. The red star denotes *E. vermicola*, the subject of this study. Table S2 lists the names and sequence numbers of the fungi involved in the construction of the evolutionary tree, which were derived from GeneBank.

Based on the genome sequencing results of *E. vermicola* CBS115803 (GCA_002778215), we identified a SNARE gene, *EvSec22*, through homology alignment. The gene has a full length of 654 bp, lacks introns, and encodes a 217-amino acid protein (Supplementary protein and genome sequence). Conserved domain prediction revealed that the C-terminal of this protein contains a conserved SNARE domain (SNC1, residues 2–195) and a transmembrane domain (residues 193–215). The SNC1 protein, a member of a highly conserved protein family, is classified as a SNARE protein that plays a crucial role in intracellular transport and secretion processes. This functional characterization strongly suggests that the *EvSec22* represents a typical SNARE-type protein associated with the secretory pathway. Homology alignment revealed that *EvSec22* is highly conserved among various fungi, including *Saccharomyces cerevisiae*, *Magnaporthe oryzae*, *Verticillium dahliae*, and *Colletotrichum orbiculare* (Figure 1A). Moreover, *EvSec22* is widely distributed among numerous fungal species (Figure 1B).

### 3.2. Optimization of E. vermicola Transformation Methods and Construction of EvSec22 Mutants and Complementary Strains

**Figure 2:**
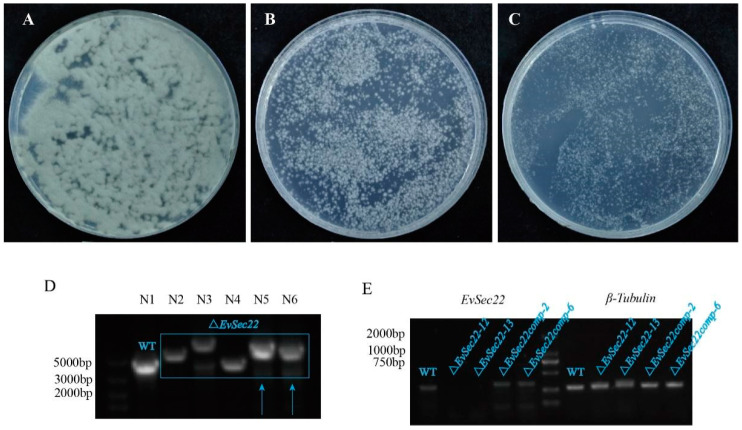
Optimization of transformation methods and mutant and gene-complementation strain construction in *E. vermicola*. (**A**–**C**) The effect of clethodim on single-colony formation in *E. vermicola* CBS115803. (**A**–**C**) Clethodim concentrations (*v*/*v*) of 0, 0.01%, and 0.02%, respectively. The photographs were taken on the 10th day after conidia plating and cultivation. (**D**) PCR amplification of the *E. vermicola* CBS115803 genome (control) and *EvSefc22* knockout mutants (blue box indicates delayed amplification bands). Among them, the bands N5 and N6 pointed by blue arrows are the correct knockout transformants, named *ΔEvSec22-12* and *ΔEvSec22-13*, respectively. (**E**) No expression of the *EvSec22* gene was detected in the mutants, while *EvSec22* gene expression was observed in both the wild-type and gene-complemented strains. *β-Tubulin* served as the internal reference gene.

The robust hyphal growth of *E. vermicola* CBS115803 hampers the selection of single-colony transformants. Clethodim, a growth inhibitor, significantly reduced hyphal growth at 0.01% and 0.02% concentrations. In this study, a concentration of 0.02% clethodim in PDA medium was employed to enhance the efficiency of single-colony isolation (Figure 2A–C).

*EvSec22* knockout mutants were screened using fluorescence exclusion (Figure S2), flank-specific PCR validation, and gene expression analysis (Figure 2D). Expression analysis confirmed the successful recovery of *EvSec22* in complemented strains (Figure 2E).

### 3.3. EvSec22 Mutants Impaired the Infectivity of E. vermicola Against B. xylophilus

**Figure 3:**
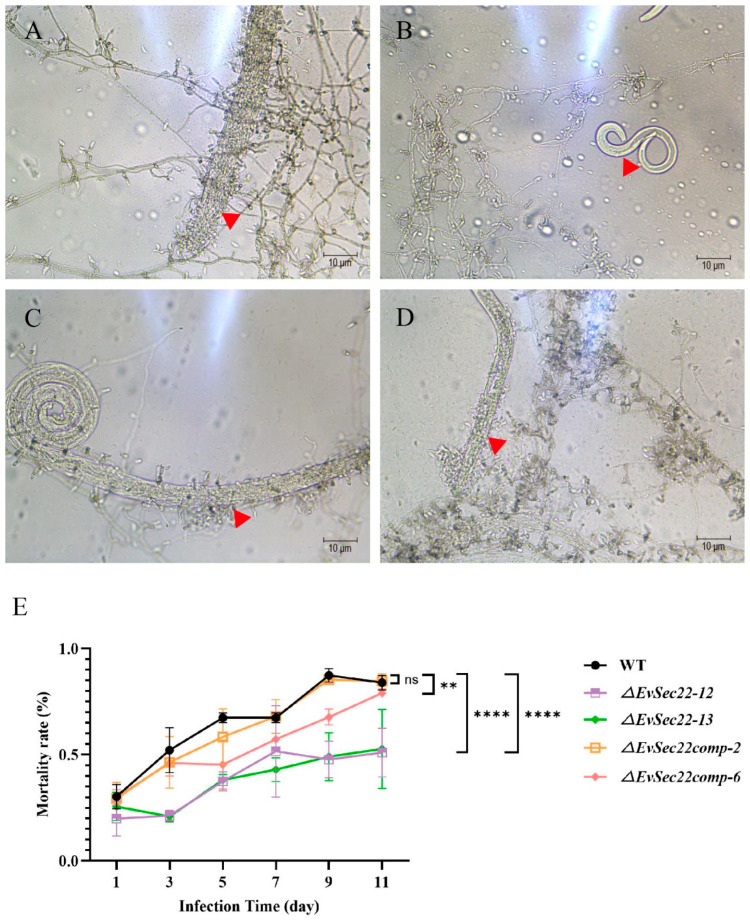
Infection assay of *E. vermicola* against *B. xylophilus.* Infection of *B. xylophilus* by the wild-type WT (**A**), *ΔEvSec22-12* (**B**), *ΔEvSec22comp-2* (**C**), and *ΔEvSec22comp-6* (**D**) strains. Photographs were taken two days post-inoculation. The red triangle represents the lunate-shaped conidia of *E. vermicola*. (**E**) Mortality rate of nematodes infected by the four strains. Compared to WT, two *ΔEvSec22* mutants exhibited significantly reduced infectivity, whereas the complemented strains restored infection capability. The experiment was repeated six times. Values represent mean ± standard deviation (SD) of six independent replications. “ns” denotes no significant difference (*p* > 0.05); “**” and “****” denote a highly significant difference (*p* < 0.01) and an extremely significant difference (*p* < 0.0001), respectively.

The results of the infection showed that after two days, nematode mortality was significantly higher in wild-type and complemented strains (Figure 3A,C,D), while nematodes in the *ΔEvSec22* group remained active (Figure 3B). Infection assays revealed that the *ΔEvSec22* strain exhibited a significantly reduced ability to infect *B. xylophilus*. After 11 days of co-culture, the nematode mortality rate of *ΔEvSec22-12* and *ΔEvSec22-13* was 51.84% and 52.67%, respectively, compared to 84% in the wild-type group. Complementation of *EvSec22* restored infectivity to near wild-type levels, with mortality rates of 84.67% and 79%, respectively. These results confirm that *EvSec22* is crucial for *E. vermicola* infectivity against *B. xylophilus*.

### 3.4. Loss of EvSec22 Leads to Slower Hyphal Growth and Hyphal Septal Spacing in E. vermicola

**Figure 4:**
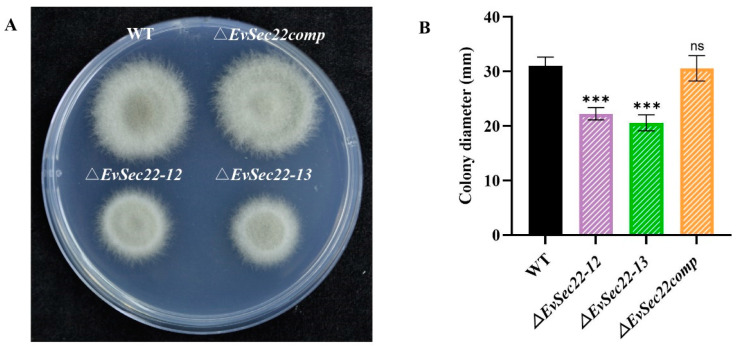
Colony diameter analysis. (**A**) Comparison of colony morphology of *E. vermicola* CBS115803, the mutant strain, and the complementation strain on PDA medium. (**B**) Comparison of colony diameters among *E. vermicola* CBS115803, the mutant strain, and the complementation strain. Since differences in growth are more visible in the same medium, WT, *ΔEvSec22-12*, *ΔEvSec22-13*, and *ΔEvSec22comp-2* strains were chosen for this experiment. Colonies were cultured at 26 °C for 8 days before imaging, and the experiment was repeated three times. Values represent mean ± SD. “ns” indicates no significant difference (*p* > 0.05); “***” denotes significant differences at *p* < 0.001.

On PDA medium, the colony morphology and color of WT, *ΔEvSec22* mutant, and complementation strains exhibited no significant differences, with all colonies displaying a grayish-white appearance and smooth margins. However, distinct variations were observed in growth rate and colony size. Deletion of *EvSec22* significantly reduced the growth rate of *E. vermicola*, whereas reintroduction of *EvSec22* into the mutant strain fully restored its growth capacity (Figure 4A,B).

We further compared the hyphal septal spacing among the different strains. The results revealed that the *ΔEvSec22* mutant exhibited a significantly reduced septal distance. However, reintroduction of *EvSec22* restored the septal spacing to WT levels in the complemented strain (Figure S2).

### 3.5. EvSec22 Deletion Reduces Total Conidia Count but Increases the Proportion of Lunate-Shaped Conidia

**Figure 5:**
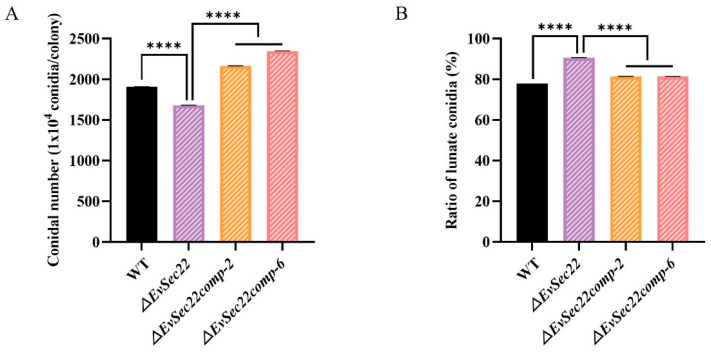
Analysis of total conidia count (**A**) and lunate-shaped conidia proportion (**B**) in PDA medium. The WT, *ΔEvSec22*, *ΔEvSec22comp-2*, and *ΔEvSec22comp-6* strains were cultured at 26 °C for 14 days. The experiment was performed in triplicate. Values represent mean ± SD. “****” denotes a significant difference at *p* < 0.0001.

Conidia play a critical role in the infection process of *E. vermicola* against *B. xylophilus*. Compared to WT, the total conidia count was significantly reduced in the *ΔEvSec22* mutant. However, reintroduction of *EvSec22* into the mutant strain significantly increased the total conidia count (Figure 5A). Surprisingly, the proportion of lunate-shaped conidia in the *ΔEvSec22* mutant was higher than that in the WT, while the *ΔEvSec22*-complemented strain exhibited a lower proportion of lunate-shaped conidia, though it remained significantly higher than that of the WT strain (Figure 5B).

### 3.6. Deletion of EvSec22 Affects E. vermicola’s Tolerance to Abiotic Stress

**Figure 6:**
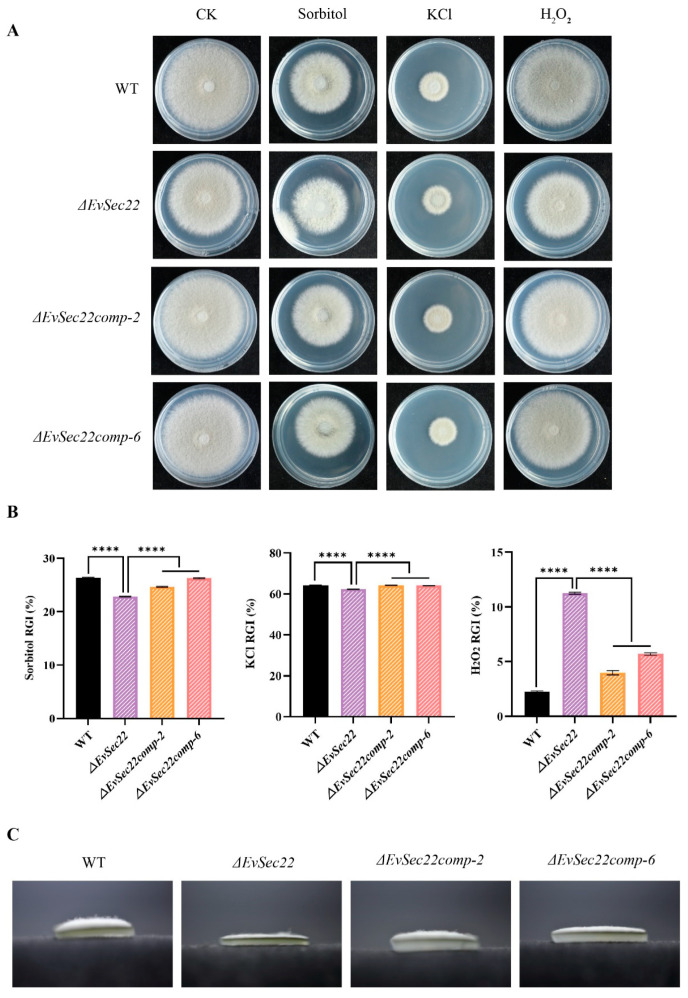
The effects of abiotic stress on the growth of different strains. (**A**) Abiotic stress conditions were simulated by supplementing PDA medium with sorbitol (1 M), potassium chloride (0.7 M), and hydrogen peroxide (1 mM). (**B**) Relative growth inhibition (RGI) was calculated based on colony diameter measurements for the WT, *ΔEvSec22*, *ΔEvSec22comp-2*, and *ΔEvSec22comp-6* strains. The experiment was performed in triplicate. Values represent mean ± SD. “****” indicates a significant difference at *p* < 0.0001. (**C**) Aerial hyphal growth of strains on sorbitol-supplemented PDA medium. Colonies were cultured at 26 °C for 10 days prior to imaging and diameter measurement.

To evaluate the role of *EvSec22* in abiotic stress tolerance in *E. vermicola*, we examined the sensitivity of four strains (WT, *ΔEvSec22*, *ΔEvSec22comp-2*, and *ΔEvSec22comp-6*) under various abiotic stress conditions, including sorbitol, potassium chloride, and hydrogen peroxide. All strains exhibited inhibited growth under these stress conditions (Figure 6A).

To quantify stress tolerance, we calculated the relative growth inhibition (RGI) based on colony diameter measurements. The *ΔEvSec22* mutant displayed enhanced tolerance to sorbitol and potassium chloride but showed extreme sensitivity to hydrogen peroxide. Reintroduction of *EvSec22* partially restored hydrogen peroxide tolerance (Figure 6B). Notably, the *ΔEvSec22* mutant formed sparse aerial mycelia on sorbitol-supplemented PDA medium, indicating that the mutant is, in fact, highly sensitive to sorbitol. Although the relative growth inhibition (RGI) values based on colony diameter showed that the *ΔEvSec22* mutant had a stronger tolerance to sorbitol (Figure 6C). These findings suggest that *EvSec22* plays a critical role in regulating hyphal growth and stress tolerance in *E. vermicola*.

## 4. Discussion

During the genetic transformation of *E. vermicola* CBS115803, we encountered challenges in obtaining single colonies due to the rapid growth of filamentous fungal hyphae. To address this, clethodim, a herbicide known to inhibit hyphal growth in filamentous fungi, was added to the medium at a concentration of 0.02%. This significantly improved the efficiency of single-colony isolation and enhanced transformation efficiency. Additionally, sodium deoxycholate was incorporated into the medium to further inhibit hyphal growth. However, we observed that its effect on *E. vermicola* was less pronounced compared to clethodim (unpublished).

The deletion of *Sec22* has been shown to impair the ability of pathogenic fungi to infect plants. In our study, the *ΔEvSec22* mutant exhibited a 30% reduction in lethality against *B. xylophilus* compared to the wild-type *E. vermicola* CBS115803. Previous research has demonstrated that VdSec22 not only regulates protein secretion but also influences conidial production [25]. Consistent with this, we observed a reduction in the total conidia count in the *ΔEvSec22* mutant, alongside an increased proportion of lunate-shaped infective conidia. This may be related to the hyphal growth state and colony size, as the proportion of newly formed hyphae at the periphery of mutant colonies was smaller. Lunate-shaped conidia play a crucial role in *E. vermicola* infection of pine wood nematodes. However, *E. vermicola* infection is influenced by multiple factors. Given that Sec22 is involved in the secretion of extracellular proteins, such as virulence-related effectors, cellulases, pectinases, and xylanases, which participate in the pathogenic process of fungal pathogens [25], this may explain the reduced infectivity of the *ΔEvSec22* mutant toward pine wood nematodes.

Our findings also revealed that the *ΔEvSec22* mutant exhibited significantly reduced hyphal growth and decreased hyphal septal spacing, which may contribute to the overall decline in conidia production. Studies on other fungi have similarly demonstrated that Sec22 is critical for hyphal germination and apical growth. For instance, deletion of *FgSec22* in *Fusarium graminearum* resulted in conidial morphological defects, including altered septation [36]. Similarly, knockout of Sec22 in *Arthrobotrys oligospora* and *Sordaria macrospora* led to reduced sporulation, lower conidia germination rates, abnormal conidia morphology, and thinner, sparser aerial hyphae [37,38]. Collectively, these results indicate that deletion of *EvSec22* impairs the growth rate, conidia composition, sporulation, and stress tolerance of *E. vermicola*, which may also explain its reduced virulence.

In response to environmental stresses and infections, organisms generate reactive oxygen species (ROS) to bolster their defenses while producing enzymes to scavenge free radicals and minimize cellular damage [39,40]. The *ΔEvSec22* mutant exhibited heightened sensitivity to hydrogen peroxide stress, suggesting an impaired ability to secrete catalase, which likely hindered its growth. When *E. vermicola* infects *B. xylophilus*, the nematode produces ROS as a defense mechanism. Efficient secretion of ROS-scavenging enzymes by *E. vermicola* could suppress this defense. The increased sensitivity of *ΔEvSec22* to hydrogen peroxide indicates a reduced capacity for ROS detoxification, potentially explaining its diminished infectivity against *B. xylophilus*.

Although relative growth inhibition (RGI) analysis based on colony diameters suggested that *ΔEvSec22* exhibited greater tolerance to sorbitol, we observed significantly reduced hyphal density in the mutant, indicating severe growth impairment. Thus, in reality, *ΔEvSec22* is highly sensitive to sorbitol. Similar findings have been reported in other studies, where high sorbitol concentrations induce osmotic stress, leading to reduced colony diameters and restricted hyphal growth [40].

In conclusion, our study demonstrates that the SNARE protein EvSec22 in *E. vermicola* CBS115803 is essential for vegetative growth, abiotic stress tolerance, and pathogenicity toward *B. xylophilus*. Building on prior evidence linking Sec22 to protein secretion in plant-microbe interactions [26], we propose that EvSec22 may similarly regulate effector trafficking during fungal infection of nematodes. To test this hypothesis, we are conducting comparative proteomic analyses between the *ΔEvSec22* mutant and wild-type strains to identify Sec22-dependent secreted proteins. Functional characterization of these candidates will clarify their roles in adhesion, virulence, or direct toxicity against *B. xylophilus*, thereby informing genetic engineering strategies to optimize *E. vermicola* CBS115803 as a biocontrol agent.

## Data Availability

The data that support the findings of this study are available from the corresponding author upon reasonable request.

## Supplementary Materials

The following supporting information can be downloaded at https://www.mdpi.com/article/10.3390/jof11040295/s1: Figure S1: Schematic representation of the knockout mutation screening strategy. Figure S2: Quantitative assessment of hyphal septal spacing in *E. vermicola* strains. Table S1: All the primers used in this study. Table S2: Fungal names and sequence numbers in the phylogenetic tree. Supplementary protein and genome sequence S1: Supplementary Methods S1.
