# Supplementary material for: EvSec22, a SNARE Protein, Regulates Hyphal Growth, Stress Tolerance, and Nematicidal Pathogenicity in Esteya vermicola
Source: J Fungi (Basel). 2025 Apr 9;11(4):295. doi: 10.3390/jof11040295 (PMC12028303; doi:10.3390/jof11040295)
Supplement: Supplementary file 1 [file jof-11-00295-s001.zip › jof-3474981-supplementary.pdf]

Journal of Fungi

Supplementary Materials

EvSec22, a SNARE Protein, Plays a Crucial Role in the Development, Stress Tolerance and Pathogenicity of *Esteya vermicola* Against *Bursaphelenchus xylophilus*

Jingjie Yuan[1], Run Zou[1], Xuan Peng[1], Yilan Wang[1], Zhongwu Cheng[1], Tengqing Ye[1], Lihui Han[1], Chengjian Xie[1]*

[1] The College of Life Science, Chongqing Normal University, Chongqing 401331, China.

ASSOCIATED CONTENT

Supplementary Figures and Tables

Figure S1. Schematic representation of the knockout mutation screening strategy.

Figure S2. Quantitative assessment of hyphal septal spacing in *E. vermicola* strains.

Table S1. All the primers used in this study.

Table S3. Fungal names and sequence numbers in the phylogenetic tree.

Supplementary protein and genome sequence S1.

Supplementary Methods S1.

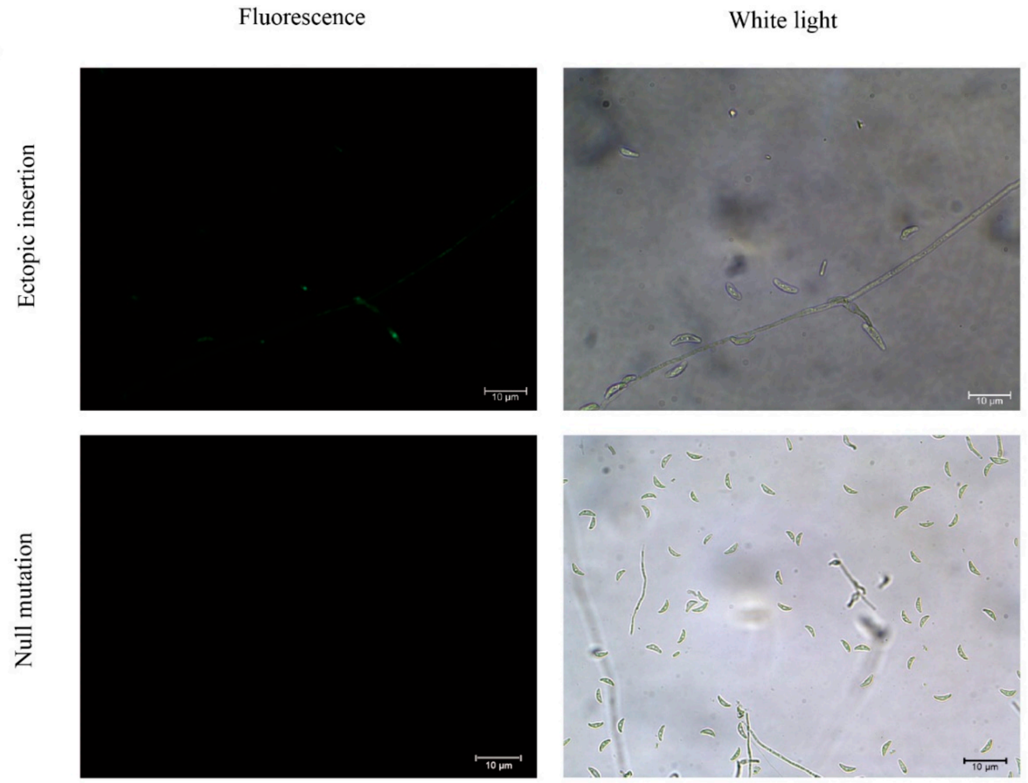

Figure S1. Schematic representation of the knockout mutation screening strategy. Ectopic insertions were detected by eGFP fluorescence emission (green), with two distinct insertion patterns observed: (i) partial fragment insertions and (ii) co-occurrence of ectopic insertions with homologous recombination events. Successful knockout mutations lacking fluorescent signal exclusively underwent precise homologous recombination without ectopic integration. Scale bar = 10 µm.

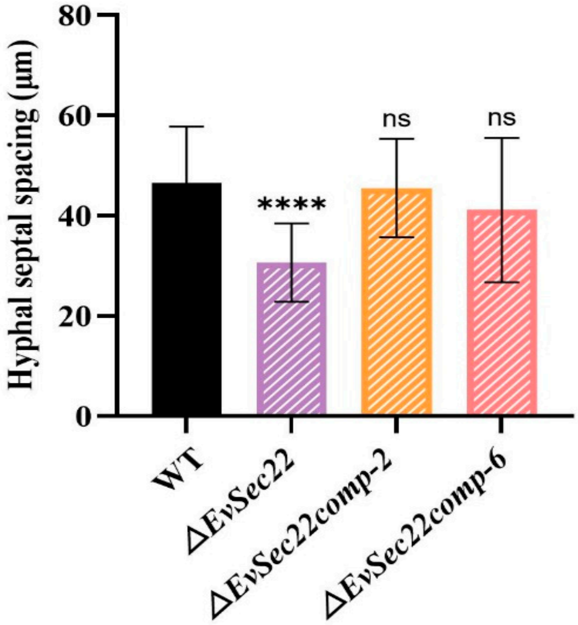

Figure S2. Quantitative assessment of hyphal septal spacing in *E. vermicola* strains. Comparative analysis of inter-septal distances among WT, *ΔEvSec22* mutant, and complemented strains (*ΔEvSec22comp-2* and *ΔEvSec22comp-6*) after 8 days of incubation at 26°C. Biological replicates (n=25) were analyzed. Statistical annotations: ns = not significant ($P > 0.05$); **** = $P < 0.0001$.

Table S1. All the primers used in this study.

Primer name	Sequence（5′-3′）
EvSec22 5coutF	CGGCCAGTGCCaagcttccaggaaggaaactacctac
EvSec22 3coutR	CTTCTGTCGACGGATCCggacgagggcgacattgaac
EvSec22 5OUTF	ccaggaaggaaactacctac
EvSec22 5f	AGAAGAGTAATCTAGcgaaggttgatggccgactc
EvSec22 5r	TTAGTGAGGGTTAATTGCGCATTTGGGTAGAGTGGATCAT
EvSec22 3f	AGTTGTTCCCAGTGATCTTCGGCGATTCTTTTGAggcggac
EvSec22 3r	ATTACGAATTGGATCaagtcgctagccaccgctac
EvSec22 3OUTR	ggacgagggcgacattgaac
2000hyg F	GCGCAATTAACCCTCACTAAA
2000hyg R	CGAAGATCACTGGGAACAACT
Evsec22 f	AGTCGCAGGTCAAGCTCATC
Evsec22 r	TGAGCTTGTCGAGGTTCTGC
Ev tub f	GCCAAGGGTCATTACACCGA
Ev tub r	ACAGGGTAGCATTGTACGGC

Table S2. Fungal names and sequence numbers in the phylogenetic tree

Serial number	Strain name
GCA_002778215	*Esteya vermicola* CBS 115803
DAA09582	*Saccharomyces cerevisiae* S288C
KLU89933	*Magnaporthiopsis poae* ATCC 64411
XP_009658406	*Verticillium dahlia* VdLs.17
BAO27797	*Colletotrichum orbiculare* 104-T
XP_003719791	*Magnaporthe oryzae* 70-15
KAK4460222	*Cladorrhinum samala* PSN324 v1.0
KXX78434	*Madurella mycetomatis* mm55
KAK3905423	*Staphylotrichum tortipilum* CBS 103.79
XP_007918533	*Phaeoacremonium minimum* UCRPA7
KUI71664	*Cytospora mali* Grove 1935
POS76942	*Diaporthe helianthi* CBS 592.81

KAH6684601	*Halenospora varia* MPI-CAGE-AT-0135
TVY47639	*Lachnellula cervina* CBS 625.97
RDW70431	*Coleophoma crateriformis* BP5796
OAA68747	*Niveomyces insectorum* RCEF 264
XP_062697517	*Neurospora Hispaniola* FGSC 10403
KAK3402964	*Sordaria brevicollis* FGSC1904
XP_014173581	*Grosmannia clavigera* kw1407
XP_016589435	*Sporothrix schenckii* 1099-18
KAK3945031	*Diplogelasinospora grovesii* CBS 340.73
XP_009223970	*Gaeumannomyces tritici* R3-111a-1

Supplementary protein and genome sequence S1.

>Evsec22 protein sequence

MIHSTQISRIDGLMFCASVDEDGATKSALSEVKSQVKLILRRLNRNAEPQASIEAAASLDIHYLIQDDVVYVCICDRSYPRKLAFTYLSDVADEFAATYPAVQRM
SPSLRPYAFMGFDTFIARTKKTYSDARASQNLDKLNDELRDVTQVMTKNIEDLLYRGDSLERMGELSSRLRDDSKKYRRAAVRINWDLLLKQYGPLAGLFFFIL
FFLWWRFF*

> Evsec22 genome sequence includes 2000bp upstream and downstream

gagagagtaggttgtcagccgccccatgcatttctatttctgtagacacggcagtgtctgccattgtctctcgtccatccatagtctggttctgctctcgagccgtcgtagattctggtcgatccggctgatcaggctgaccagactgatccgtc
aggctgtctacaggacgtgagtaggctggacggtaggcctctggcagctggatattccactcgcgctcaaacgtctcggcaataaactggtcaaagatgccgctcttgcggagttcgtgcgcggcataggcctcggcgagccatttcttg
tcgtgaaagccagtccggaggtcgttctgatagcgggcacagtcgtgccggaagctgtcgtcgttcttgagcgacgcaatatccggccgggcctggtcggtgtcggcatcgacaatgtgccgcccgctgggaaagagcaggagcacct
ctctctggtcctctggggtgagtgcctgccaggcctgaggattgcagaggagggactgccacgttagccttgcgtctgcagaatccaagccagaatccaagccaggaaggaaactacctacccggatgtcgtagttggccagcggcga
tttggcattggagagaacaaattccggctgccaccgggcggcctcgcgcttgcggcgaaggttgatggccgactccgacggcgacttggccggccggggcgcttcctcgccaaaagagctcgatcgcttgcgagcgattcgtctgcca
gagcgagacactgtgacgggaggcgaagaaggcaaaggcggcggagacggcgaaggaggcatgcctgagacgagagtgtctgttagcagagagagaaagagggaaaagagaagaaaaaaaagagtgctcttcacctggag
gagggggcttccgatctggcagacggggagacggatccgcagacatggacgacgtcgtgacgggtgcctctgttctgggtggcatggcatcagacacagtctcgtgggcagcttcagcggggcaggaaacaggaagagggtgag
agtcttgtgccttgccggccgagggagagggggggtgaaggagagctggggatctctggtggcaagcgagtcaaggaagaaaaaatctgggaatgcggcggaggaaaagggtgagcgaggcagttcatgtcctcgtcaaactggt
tggaagagagggccgaggacagggggaggaggaggcgctcgagtccattgtgatagagaacagaaaagaaatttcagaaaatgaaaggaactctctggcgaagaagaaatgccagaatttttatgacatggcagtggactaga
gaagagtattaaaaggcaaaagacaaaagacaaagacaaaaggaaaaaggaaaaaggaaaaaagaacggcaatttgtctcgctctatagtttggctaaaaatggacaagaatgtcatgtgtggcgaagatcgcagagacggcga
agatcgcgtacacgacagcgcaagcagacacggtcggaaggactcttgtgttgtttttctttccccaggtgagaatgggctcgactagctctcaagcttccgtcacaaaggagctctcgcgaggctggtgtcacgggtgatcgcagtacta
gccagaagagcgagtcacagccacaatggagaaagacaagccacatgcttgcttatataatacaatcggttggccagccttgtgaaatgtggagttttggcctctttgtctttgctgcagaaacggccactttatatttttatttttatttttgga
aatcaggggcagcaaccaaccaaccttttcgcagggagccgcaccagacgacaaacgagcctagacgaccctagacgacaccattggctcttcctcttactttgtatcctcacaccgactgtccatctcgtcctcgtcctcatcctcgtcctcg
tcttcatccagccacgtcaccctttttttgccgactgacagcttcgacctcgacatcgactccctccccctcaagATGATCCACTCTACCCAAATCTCCCGCATCGACGGGCTCATGTTCTGCGCCTC
GGTCGACGAAGACGGCGCCACCAAGTCGGCCCTCAGCGAGGTCAAGTCGCAGGTCAAGCTCATCCTGCGGCGGCTGAACCGCAACGCAGAGCCCC
AGGCGTCCATCGAGGCGGCCGCCAGCCTTGACATCCACTACCTCATCCAGGACGACGTCGTCTACGTCTGCATCTGCGACCGCTCCTACCCCCGCAA
GCTAGCCTTTACCTACCTCTCCGACGTCGCCGACGAGTTTGCCGCGACCTACCCGGCCGTCCAGCGCATGAGCCCGTCGCTGCGGCCCTATGCCTTTAT
GGGGTTTGACACGTTTATCGCGCGCACGAAGAAGACGTACAGCGACGCGCGGGCGTCGCAGAACCTCGACAAGCTCAACGACGAGCTGCGCGACG
TCACGCAGGTGATGACCAAGAACATTGAGGATCTGCTCTACCGCGGCGACAGCCTCGAGCGCATGGGCGAGCTCAGCAGCCGCCTGCGCGACGACA
GCAAAAAGTACCGCCGCGCCGCCGTGCGCATCAACTGGGACCTGCTGCTGAAGCAGTACGGACCTCTAGCCGGTCTCTTTTTCTTTATCCTCTTCTTTC
TCTGGTGGCGATTCTTTTGAggcggacggggcgtggtaacgtcctggttttcccttgtttccttgtttcctttttgcttcttctttttctcgagctcagaaattgtacggactgtacattaacattagcaacattggcgttttgggtg
gagggggagagcggcggacccgttggatgggctagaattgttttcctgtgaaaaaaaataaagaaaaaaaagaatatccaatcacaatctgtgctcctagacatcatctacacattggtatcatggctgtttgccgctgtcttttctatttctt
gctcagtcttgctcctgtggcgcaagagggatgtaaaatgtcataagagtcgtatcgaaacgccacgcaatgcctcgcccatcaaacatccaaacatcaaacaatacagacgacacggtatacaaaagactgtaccccaaaagtggaac
aagttcctccatgtctgtaaaaaaaacgaaaaaaaaaaaaacaggattttcctccattctcccgagaacgcccgctggatataaaacctcgtgtccaagactgtaagacaataacattaggaaaacaatagagggataaaaaaaggtggtt
gtaatacaaaacagcttgcacgttgtctgccgtctctcgtgctacattaatgtgcagttggtggatggtgtggggagggcgggaaggggcagcgccgtgtggtaccactcgaggtgttcttgaaaggcggcgagactgggaaagccaa
actcgtcctgggcgtaccgtgtgtggtcgccttcggccgtgtcgtagtacatgtagtcgtcgcagtaaggacaccagagccgagcctcccagtcgccaccggtggggggcaggtggttggcaatggcgagcggcgcaaagacaatgg
tacgctccttttcctggctggaaacggccgtggtggtggcttgccactgcggatagccgtccgccgacttgggcgggacgggaggcagtgtcgagttggccgaggcggccgaggcggccgaggggcggatatcggcaggatggg
cggtagggacgaggttttggtcgtcaaggacagcgaggtgggacgcgtcatcgagtctgcgggggggcacgcgagaggtgggcaggcggtagacgtcttcccagacaatgtgaccgtcgcagtagtcacagacaaagacgatgtcg
cggtcgccaaagcgctcaaaggagccaatggcattgcagtcgggcacgaggtcttcgaggctgggccggcggggcttgtcgtacggggacgcgtgggagtagggcggcaaggaggagtcttgcatggccgaaacggccgctgcg
ccgagagccgtggcggtagcggtggctagcgacttgccgtcgagcgagacgggcgtaccgggcagaggcgtggccgtggctgtggccgtggccgtggcggagacgagggtcgaggctggcaggccggcgagttcaatgtcgcc
ctcgtcctgacaactgtagctggcctggcgactgtggctgagctgttcgcggccggcatagcgctcgtcctctcgctcttgcatccgcctctgctgcagtttccaagtgggcacgcgctggagggtcggccgtcgcgaagagttgggcgac
tggcgcgagaggcgtctcttctgctgctgctccttttgttgctgttcttgcttcagctgctcttgctggctgtgcttctcttgctgctcgcgcagacggcggtagacgaggtaggcgccgagacagcgggcgccgcttccgccgacctcgcgtt
cggcgttgaagatgcggcgggcagcacggcactgggctcggtggagttgtcggcggaggtcgacgacgtcgcgggtctgcagaggactaatgggaccgccggagcgtagccagtctttgagggtctgggtgtggctgtcaaacttt
gctcgcagagctcgaagctcttcgagagactcgcctagactgttagcgggccagaagaagacggcgaagagacaaaatgacaaggaaaaaaacaca

Supplementary Methods S1

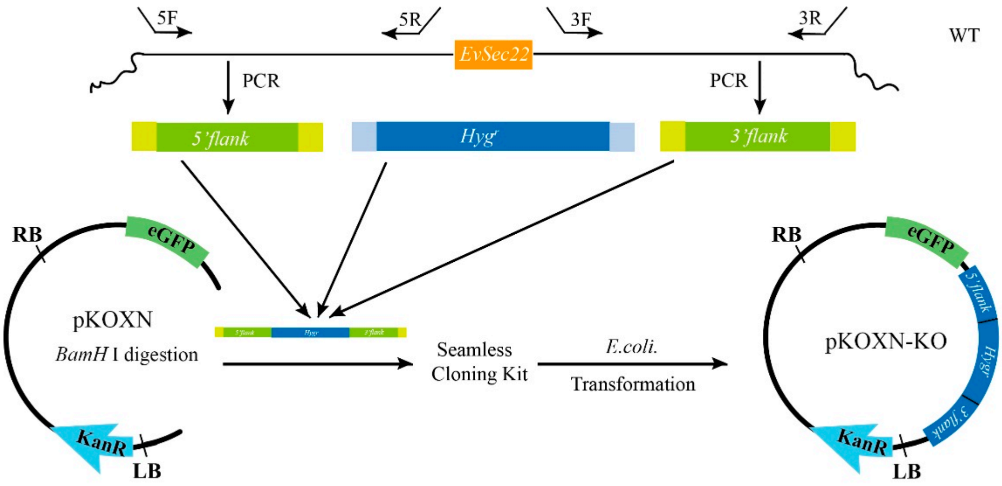

Construction of gene knock-out vectors.

1. The 5' and 3' fragments were amplified from the genomic DNA of *E. vermicola* CBS115803 using the *EvSec22*-5F/5R primer and *EvSec22*-3F/3R primer, respectively, and Hyg^r (HygF/R) was amplified from the vector psilent1, and these three fragments were subsequently fused by PCR.

2. The vector pKOXN was knocked out with *Bam*H I monoenzyme digestion, and then ligated to the fusion fragments using 2×Seamless Cloning Kit. The obtained pKOXN- *EvSec22* construct was transformed into *E. coli*. The *EvSec22*-5F and *EvSec22*-3R sequences in the construct contained fragments homologous to the pKOXN- *EvSec22*.

3. The knockout vector pKOXN- *EvSec22* was transformed into Agrobacterium agalactiae AGL1, followed by ATMT-mediated transformation as described in the article[30].

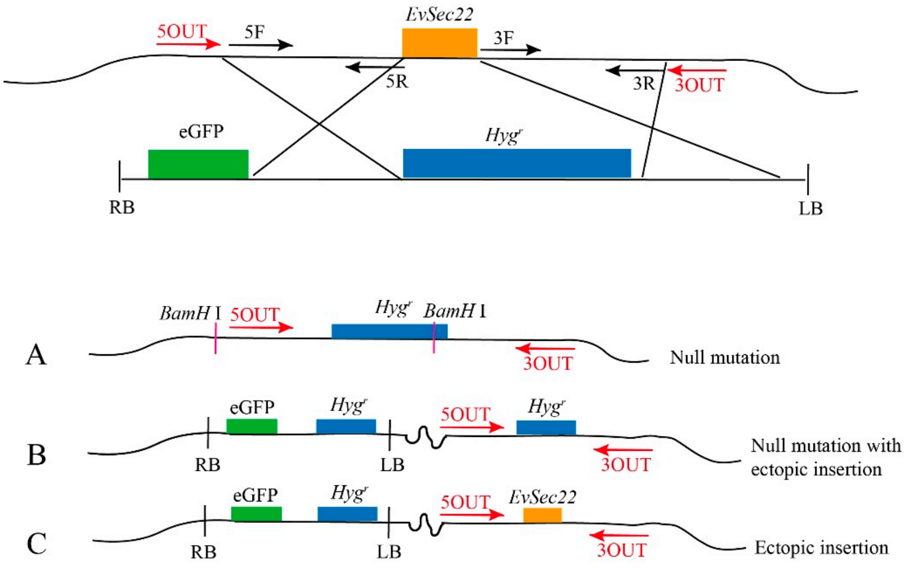

Identification of null mutant transformants.

1. Transformants are screened for GFP fluorescence under a microscope. Strains without GFP fluorescence will be retained for further analysis. *Homologous recombination produces three types of transformants: **A:** null mutant; **B:** ectopically inserted null

mutant; **C:** ectopically inserted transformant. **B** and **C** have GFP fluorescence because the GFP gene is contained in the T-DNA fragments (RB and LB). The null mutant (**A**) has no GFP fluorescence because the GFP gene is eliminated in the null mutant.

2. Primers 5OUT and 3OUT are used to amplify the gDNA of the transformants. the primers for the null mutant should be longer than the WT or ectopic insertion into the transformant.

3. Reverse transcription (RT)-PCR was performed to confirm the absence of *EvSec22* mRNA in the null mutant.
